# Retroviruses and Cancer: Coevolution and Genetic Exchanges Between the Viral and the Host Genomes

**DOI:** 10.3390/biology15120972

**Published:** 2026-06-21

**Authors:** Xuhua Xia

**Affiliations:** 1 Department of Biology, University of Ottawa, 30 Marie Curie, Ottawa, ON K1N 6N5, Canada; xuhua.xia@uottawa.ca; 2 Ottawa Institute of Systems Biology, Ottawa, ON K1H 8M5, Canada

**Keywords:** retrovirus, cancer, horizontal gene transfer, host–parasite coevolution, genetic exchange, endogenous retroviruses, *v-Src*, *v-sis*, syncytin, suppressyn

## Abstract

Retroviruses, after their genomes are integrated into the host genome, replicate through host cell replication. In this hitchhiking phase, their only way of increasing their fitness is to encourage the host cell to have unregulated, rapid cell replication. The *v-Src* gene in avian sarcoma virus and the *v-sis* gene in the simian sarcoma virus were originally mined from the host genome by the virus to increase host cell replication rate, with the corresponding host cellular counterparts *c-Src* (non-receptor tyrosine kinase) and *c-sis* (platelet-derived growth factor). The resulting out-of-control replication ultimately would lead to cancer. The battle between the host and the retroviruses left many retroviral corpses known as endogenous retroviruses, and the host occasionally domesticates retroviral genes. The syncytins (whose fusogenic function is crucial for the trophoblast fusion and the formation of a syncytium during placenta morphogenesis) and suppressyn (which serves the dual function of regulating syncytialization and host resistance against retroviruses) are examples of successful domestication. Syncytin-1 and suppressyn have each been “domesticated” independently multiple times by different mammalian lineages. Molecular phylogenetics is an essential tool for tracing the evolutionary trajectories of such genetic exchanges between retroviruses and their hosts and for determining the direction of the genetic exchange.

## Simple Summary

Retroviruses and their hosts have exchanged genes throughout evolution. Some retroviruses have acquired host genes that promote cell growth, allowing infected cells to replicate rapidly and increasing viral fitness. Classic examples are the *v-Src* gene of Rous sarcoma virus and the *v-sis* gene of simian sarcoma virus, both derived from normal host genes. While advantageous to the virus, these captured genes can drive uncontrolled cell proliferation and contribute to cancer. Genetic exchange can also occur in the opposite direction. After retroviruses become permanently integrated into host genomes as endogenous retroviruses, some of their genes may be co-opted by the host and acquire beneficial functions. Examples include syncytin-1, which is important for placental development, and suppressyn, which helps regulate placental cell fusion and can protect cells against retroviral infection. This paper refines evidence for both host-to-virus and virus-to-host gene transfers, with emphasis on how molecular phylogenetics can be used to infer the direction of transfer and reconstruct evolutionary history. These examples illustrate a long-term coevolutionary relationship in which retroviruses and their hosts have repeatedly exchanged genetic material, shaping both viral pathogenicity and host biological innovation.

## 1. Introduction

The link between retroviruses and cancer dates back to 1910, when the cell-free extract of a chicken tumor caused by Rous Sarcoma virus (RSV, a retrovirus) was shown to be oncogenic [1,2]. As the oncogenic property of cancer cells can be transmitted from parental cells to daughter cells, the genetic material of the cells was most likely modified by the virus. This hypothesis became mechanically plausible when the reverse transcriptase was discovered in both RSV and in murine leukemia virus [3,4], enriching the central dogma. In particular, the identification of the *v-Src* gene as an oncogene [5] and the subsequent studies have established a close link between retroviruses and cancer [6,7,8,9], especially between cancer and human endogenous retroviruses [8,10,11].

A question naturally arising from these discoveries is how retroviruses cause cancer. An answer was hinted at by the discovered sequence similarity between an oncogene, *v-sis*, and the platelet-derived growth factor, PDGF [12,13]. This finding not only resulted in PDGF and several other growth factors being recognized as drug targets against cancer [14,15,16], but also led to two new lines of thinking. First, the viral transforming factor may work simply by changing the regulated expression of a growth factor to constitutive expression, suggesting growth factors as targets for anti-cancer drug development. Second, any factor modulating gene expression patterns of growth factors or cell replication gatekeepers can potentially contribute to cancer. This new conceptual framework of cancer biology contributed to the progress of mechanism-based anti-cancer drug development in the following years [17,18,19].

Such findings ushered in an evolutionary theory on retroviruses and cancer that explains why viral agents that cause cancer are often growth factors or regulators that encourage cell growth and proliferation. When the genome of a retrovirus is integrated into the host genome, its fitness depends on how fast the host cell can replicate. It is therefore beneficial for retroviruses to evolve molecular mechanisms or growth factors to encourage the rapid growth and proliferation of host cells. Similarly, bacteriophage in its lysogenic phase can also help its bacterial host cell to grow and replicate [20,21].

Many retroviral oncogenes have cellular counterparts, e.g., the retroviral *v-Src* has a cellular counterpart *c-Src* [22], and the retroviral *v-sis* has multiple cellular counterparts (e.g., *PDGFA*, *PDGFB*, *PDGFC*, and *PDGFD* in human) [12,13,23]. One naturally would ask which one is ancestral. This harks back to the old but continuing controversy surrounding the virus-early and virus-late hypotheses [24,25]. If the viral world antedates the origin of cellular organisms, then viruses could potentially serve as the source of many genes for cellular organisms. In contrast, if viruses arose long after the diversification of the cellular organisms, then most likely viral genes were derived from cellular organisms.

When sequence or function similarity is observed between a retroviral gene and a host gene, two alternative hypotheses of gene origin need to be discriminated. The first is the host-first hypothesis, i.e., the gene originated first in the host but was co-opted by retroviral lineages. The second is the retrovirus-first hypothesis, i.e., the gene originated first in retroviruses and was “domesticated” by the host. Take the discovery of human syncytin-1, for example. Sequence similarity between syncytin-1 and the retroviral env protein was documented, as was the functional similarity between the two, i.e., both cause cell–cell fusion [26]. However, sequence or functional similarity alone is insufficient to discriminate between the two hypotheses. To substantiate the retrovirus-first hypothesis concerning syncytin-1, one could check if the host gene is flanked by retroviral sequences. Specifically, if the *ERVW-1* gene (encoding syncytin-1) is located at the position of the retroviral *env* gene within a remnant retrovirus. However, such evidence is often difficult to establish because mutations often obliterate the retroviral remnants.

Molecular phylogenetics provides decisive tools for identifying ancestry between the viral and cellular counterparts and a conceptual framework for inferring the evolutionary trajectory of retroviral and host genes participating in the genetic exchange. Retroviral genes such as *v-Src* and *v-sis* are clearly derived from vertebrate cellular genes, but many other cellular genes, such as *ERVW-1* encoding Syncytin-1 and *ERVH48-1* encoding suppressyn, were derived from retroviruses.

## 2. Materials and Methods

Retroviral genomes of Rous sarcoma virus (RSV) strains and simian sarcoma virus (SSV) strains were downloaded from GenBank in GenBank flat files (.gbff files). The *v-Src* gene in RSV and the *v-sis* gene from SSV were extracted using DAMBE v7.5 [27]. The orthologous genes of *v-Src* and *v-sis* were extracted from sequenced genomes of individual species. The *ERV-W-1* gene encoding syncytin-1 and the *ERVH48-1* gene encoding suppressyn were also downloaded from the NCBI Orthologs database. All coding sequences were parsed using DAMBE from downloaded .gbff files. Only fully resolved sequences (i.e., with no unresolved bases) were kept in the analysis. Also removed are coding sequences with an embedded in-frame codon. For genes with alternative splicing isoforms, only the longest one was kept as a representative. Genes that contain in-frame stop codons were eliminated. Genes from hybrids, such as between *Bos taurus* and *B. indicus*, are also eliminated.

Sequences were aligned by using MAFFT v.7.511 [28] with the slow but accurate option (L-INS-i). We have used post-alignment adjustment [29] as well as the three-way alignment to improve the initial tree for multiple sequence alignment [30], but they do not improve the sequence alignment consistently, based on the weighted sum-of-pairs score [29,31,32]. Phylogenetic reconstruction was performed with PhyML v3.1 [33], with the LG empirical model used for aligned amino acid sequences and the GTR model used for nucleotide sequences. The best tree was chosen from the best nearest-neighbor interchange (NNI) and subtree-pruning and regrafting. The option of simultaneous optimization of tree topology, substitution rates, and branch lengths was chosen for parameter estimation.

Protein isoelectric point was calculated with DAMBE, using a sliding window of 80 residues and a step length of one. This is needed to understand the inter-domain electrostatic interactions in proteins, especially the regulation of c-Src.

Both *ERVW-1* and the *ERVH48-1* genes represent horizontally transferred genes in mammalian species. Tracing horizontal gene transfer events typically would require an approximated species tree reconstructed by using informational gene sequences (e.g., genes used in DNA replication, transcription, and translation) that are rarely found to participate in horizontal gene transfer events [34]. However, for vertebrate species, especially for mammalian species, the phylogenetic relationship is well established, and the hierarchical relationship at the Integrated Taxonomic Information System (ITIS: https://www.itis.gov/) is taken as an approximation of the species tree.

## 3. Results

### 3.1. Retroviral Genes Derived from Host Cellular Genes

From a viral perspective, transduction is a major mutation event. Most of such events are probably deleterious, but occasionally, some might be beneficial. Viral genes *v-Src* and *v-sis* represent products of such beneficial transduction events.

#### 3.1.1. *v-Src* and Its Origin in Rous Sarcoma Virus (RSV)

**Figure 1:**
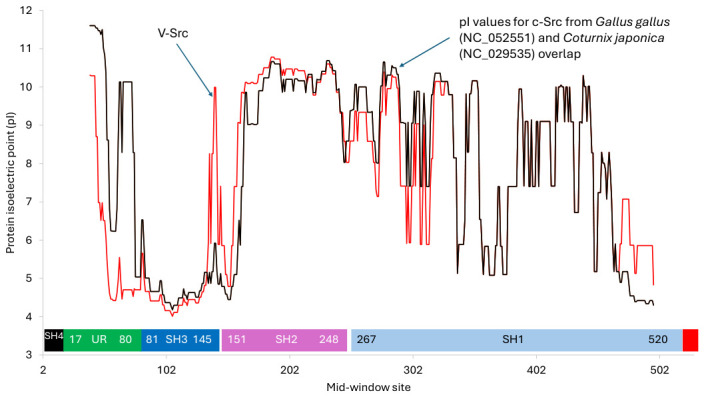
Domain structure (above the *X*-axis) and protein isoelectric point (pI) profile of c-Src from two avian species (chicken and quail) and v-Src from Rous sarcoma virus. pI values are plotted along a sliding window of 80 residues. A point at *X*-axis of 102 is calculated from window spanning sites 63 to 142. The chicken and quail c-Src proteins have 533 residues structured into four domains (SH1, SH2, SH3, and SH4, as well as a unique region (UR) between SH3 and SH4. The two numbers at the two ends of each domain indicate the approximate domain boundaries. The C-terminus of v-Src (red) is nearly neutral in contract to the acidic C-terminus of c-Src.

*c-Src* (the cellular counterpart of *v-Src*) is a tyrosine-protein kinase [35] with four relatively conserved domains numbered from the C-terminus back to the N-terminus: SH1, SH2, SH3, and SH4, and a variable unique region between SH3 and SH4. The first methionine undergoes the N-terminal methionine excision, which occurs in nearly all proteins from bacteria to mammals when the first methionine residue is followed by a small and nonpolar residue such as glycine or alanine [36,37,38,39,40]. The second residue in c-Src is a glycine, which is myristoylated [41]. Myristoylation is implicated in protein subcellular relocalization [41], apoptosis [42,43], signal transduction [44,45], and the virulence and colonization of pathogens [46,47,48,49,50]. Myristoylation occurs only on a Gly residue [41], so the glycine residue at the second position is invariant in all c-Src and v-Src proteins in amphibians, reptiles, birds, and mammals. Myristoylation is essential for v-Src to attach to the plasma membrane, and this attachment is essential for the transforming activity of v-Src. Mutations that prevent myristoylation abolish membrane localization and strongly reduce the oncogenic activity of v-Src.

c-Src is an on–off switch regulating many cellular processes [51,52], including cell proliferation and development of cancer [53,54]. The molecular switch is mainly mediated by the phosphorylation state at Y527, as revealed by protein structure and other experimental studies [55,56,57,58,59]. As we can see from the pI profile (Figure 1), the C-terminus of c-Src has low pI values (because of having multiple aspartic and glutamic residues) and is expected to be strongly negatively charged under physiological conditions. In contrast, SH2 is strongly positively charged. The negatively charged C-terminus reaches out to the positively charged SH2, embedding the catalytic tyrosine kinase domain in a closed inactive state. When Y527 is phosphorylated, the C-terminal becomes even more negative and binds to the positively charged SH2 even more tightly. To activate the tyrosine kinase, Y527 is dephosphorylated [60,61], weakening the binding between the C-terminus and SH2 of c-Src. The kinase activity is further enhanced by the autophosphorylation of Y416 in c-Src. I should mention that the residue numbering follows that of the chicken c-Src. However, the chicken c-Src is expected to have two splicing isoforms, one with a RKVDVR insertion and the other without. The c-Src without the insertion has been used in traditional studies, so the numbering follows this shorter splicing isoform.

The electrostatic interaction between the C-terminus and the SH2 as a mechanistic explanation of the genetic switch can also explain why v-Src is in a constant on-state. v-Src misses 18 residues at the C-terminal of c-Src and does not carry as much negative charge as the C-terminus of c-Src. As one can see from Figure 1, the C-terminus of v-Src has an average pI value close to seven, i.e., and is expected to carry little negative charge. This C-terminus, therefore, cannot interact favorably with the positively charged SH2 to lock the tyrosine kinase domain into an inactive state.

**Figure 2:**
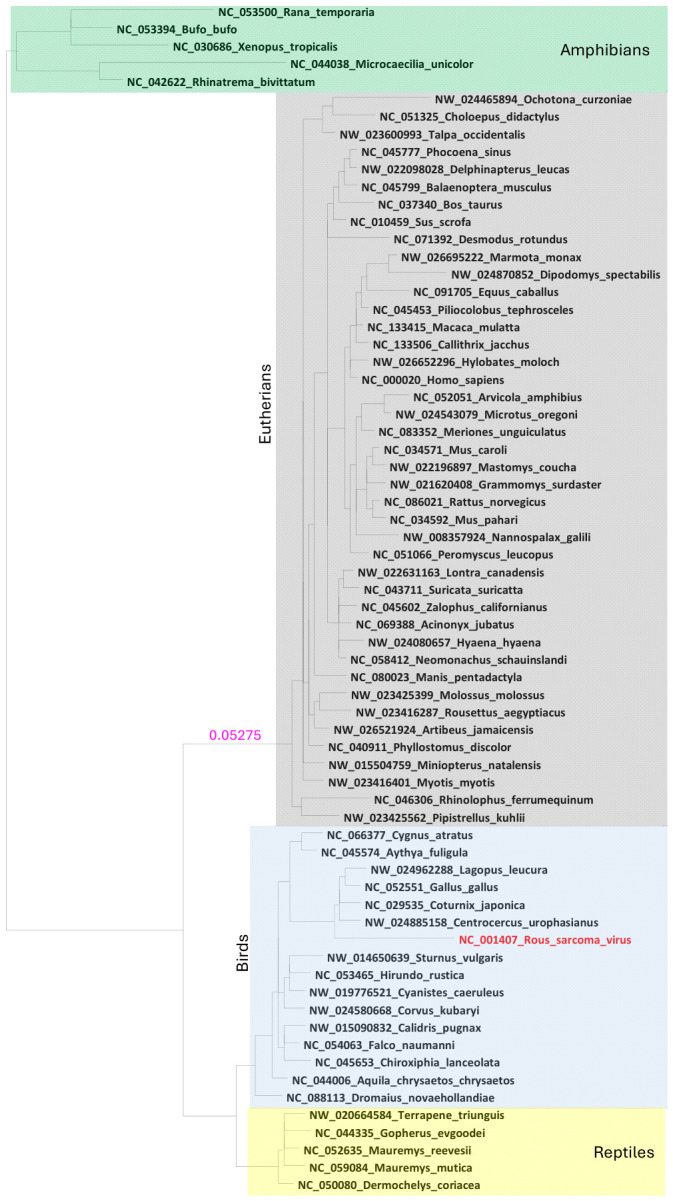
Phylogenetic tree of *c-Src* from major tetrapod taxa and *v-Src* represented by Rous sarcoma virus (colored red). The phylogenetic reconstruction is based on aligned amino acid sequences corresponding to sites 1–516 of *c-Src* from the chicken (NC_052551, *Gallus gallus*). Major taxonomic groups are shaded and labeled. Sequence names are in the form of “GenBank accession”_”species name”. One branch length (0.05275 in pink) was labeled to serve as a scale bar.

As for the two hypotheses on the origin of the *Src* gene, i.e., host-first or retrovirus-first, a phylogenetic tree is often sufficient to support the host-first hypothesis. *c-Src* is present among all major vertebrate lineages long before the origin of *v-Src* (Figure 2). The tree did not include shark and fish species, but *Src* is known to be present in these vertebrate lineages [62]. The phylogenetic tree is based on the amino acid sequences corresponding to sites 1–516 of the c-Src protein from the chicken (*Gallus gallus*, NC_052551). The chicken *Src* gene has 533 sense codons. The first 516 residues of the chicken Src are highly homologous to the RSV’s v-Src, but the last 19 codons of vertebrate *c-Src* (18 sense codons plus the stop codon) exhibit no homology [63,64] to *v-Src,* i.e., the two were not alignable. The C-terminal segment of c-Src contains a tyrosine residue at site 527 that, when phosphorylated, inhibits the kinase function of the protein. Because v-Src is a truncated version of c-Src and consequently lacks this inhibitory phosphorylation site, it is constitutively active as opposed to c-Src, which is strongly regulated [65,66].

The (*c-Src* + *v-Src*) gene tree (Figure 2) is concordant with the generally accepted relationship at ITIS (https://www.itis.gov/). It shows clearly that *c-Src* originated long before *v-Src,* which is only recently derived from chicken or species closely related to chicken (Figure 2). This is consistent with previous interpretations and reconstructions of the recombination events that gave rise to *v-Src* [67]. The tree in Figure 2 suggests that RSV may not be derived from *G. gallus,* but instead from the common ancestor of phasianids (Figure 2). However, the host-first hypothesis is clearly supported.

It is implicitly assumed that the *v-Src* gene in RSV was derived from a recombination event between a chicken’s *c-Src* region and an avian leucosis virus (ALV) [68], for three reasons. First, RSV infecting chickens contains *v-Src* [68,69]. Second, although *c-Src* is present in all tested vertebrate species, the chicken *c-Src* appears more closely related to the *v-Src* [62], with there being no *c-Src* sequences from other phasianid species in the early studies. Because *v-Src* is a 3′-truncated version of *c-Src,* it does not include the last 19 codons (18 sense codons and one stop codon) of *c-Src.* One would therefore expect the truncated *v-Src* to expand its 3′ end into downstream viral sequence until an in-frame stop codon is encountered. As it happened, the 3′-truncated *v-Src* expanded 12 sense codons downstream to encounter a TAA stop codon.

**Figure 3:**
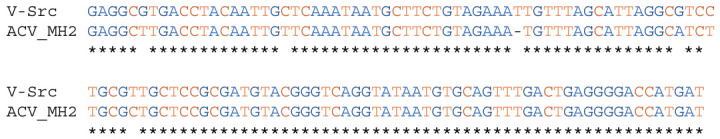
Homology between the 3′-UTR of *v-Src* (four nucleotides after the stop codon) and the noncoding region upstream of the 3′ long-terminal-repeat of avian carcinoma virus (ACV MH2) (NC_001402, sites 2064–2185). The two purines share similar colors, so do the two pyrimidines. Identical sites are indicated by “*”.

Given this scenario of *v-Src* origin above, one would expect the *v-Src* gene to be flanked by ALV sequences. The RSV sequence upstream of the *v-Src* is indeed homologous to that of ALV. However, the RSV sequence downstream of *v-Src* is not homologous to any sequenced ALV genome. Interestingly, four nucleotides after the *v-Src* stop codon, the RSV sequence is homologous to the noncoding region of avian carcinoma virus MH2 (ACV MH2), just upstream of the 3′ long terminal repeat in the ACV MH2 genome (Figure 3). This suggests a more complicated scenario of *v-Src* origin. That is, a 3′-truncated *v-Src* might have been co-opted by ACV MH2 first, and this *v-Src* and the downstream sequence of ACV MH2 origin were then recombined into an ALV to become RSV. A problem with this hypothesis is that no *v-Src* has been recorded in any ACV strains, which carry different oncogenes such as *v-mil* and *v-myc*.

I should mention that the observation of a gene tree consistent with the species tree does not imply that the gene did not originate through horizontal gene transfer. For example, many eukaryotic genes are of bacterial origin, being acquired after the progenitor of the mitochondria, most likely a Rickettsia-like bacterium, was internalized into a host cell [70,71,72,73]. Representatives of these eukaryotic genes of bacterial origin include triosephosphate isomerase [74]. The triosephosphate isomerase gene tree is largely concordant with the generally accepted species tree [74,75,76]. Such concordance between the gene tree and the species tree indicates the lack of horizontal transfer events along the evolutionary lineages in the tree, but does not imply that triosephosphate isomerase did not originate through horizontal gene transfer at a time point before the common ancestor of the lineages in the tree. Similarly, the tree in Figure 2 only indicates that the *c-Src* gene did not participate in horizontal gene transfer after the origin of vertebrates, but cannot exclude the possibility that *c-Src* may have originated through horizontal gene transfer before the origin of vertebrates.

#### 3.1.2. *v-sis* and Its Origin in Simian Sarcoma Virus (SSV)

The viral *v-sis* gene encodes a PDGF-B-like protein [12,13,23,77,78] which competes with the host PDGF and constitutively autoactivates the PDGF receptor of the cell where it is expressed [79,80,81,82]. In contrast, the cellular PDGF (or *c-sis*) is a highly regulated growth factor participating in normal cell growth and development [81], although mutations in the host PDGF or the loss of its regulation could potentially contribute to cancer as well [83,84,85,86]. In cells that express the same level of v-sis or PDGF, the former undergoes cancer transformation, but the latter does not [81].

**Figure 4:**
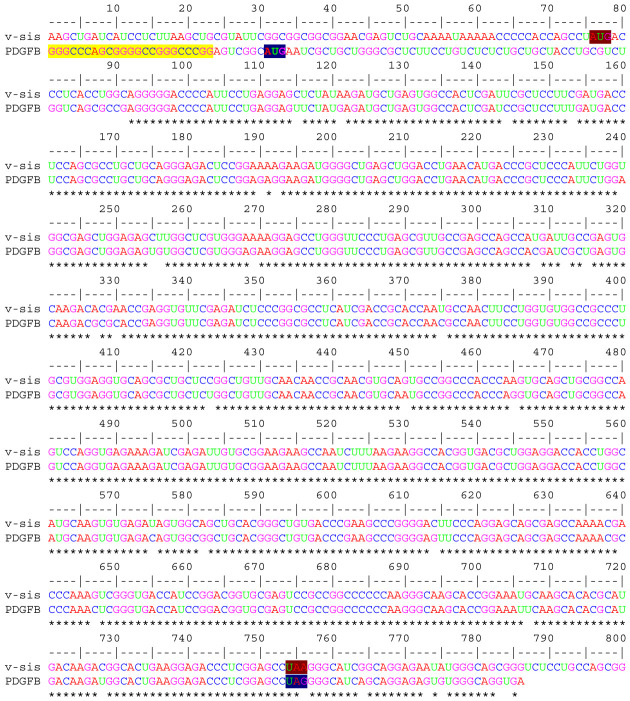
Alignment between *v-sis* from woolly monkey sarcoma virus (Accession NC_009424.5) and *PDGF-B* from *Saimiri boliviensis* (Accession NC_133469). The yellow-shaded region is the GC-rich regulatory sequence. Also shaded are the start and stop codons. Identical sites are indicated by “*”.

The alignment between the viral *v-sis* and the cellular *PGDG-B* (Figure 4) shows that the viral gene does not have the upstream GC-rich regulatory sequence and the coding part of the first exon in the cellular *PDGF-B* gene [87]. The first exon in *PDGF-B* encodes a signal peptide that is cleaved off to produce the functional *PDGF-B* chain. Another difference not shown in Figure 4 is that the cellular *PDGF-B* gene has an AU-rich 3’ UTR that is also missing in *v-sis* [87]. For phylogenetic reconstruction, the first 91 nucleotides in Figure 4 were removed, and so were the nucleotides after the stop codon.

The partial *PDGF-B* gene was incorporated in the middle of the viral *env* gene and destroyed the function of the *env* gene, rendering SSV replication-defective. This is not unique in SSV and occurs in RSV as well. The reference genome for RSV (NC_001407) happens to be from the Prague-C strain of RSV, which has the co-opted *v-Src* inserted in a viral genomic location that does not disrupt any of the functional genes, including *env.* The genome annotation does not include strain information, but UniProt (https://www.uniprot.org/taxonomy/11888, accessed on 17 October 2025) annotated a Prague-C RSV that matches the RSV reference genome. However, other RSV strains, e.g., RSV-29 [88], can have the *env* gene disrupted by *v-Src* and become replication-defective. Such replication-defective strains would need a helper virus for replication. In the case of SSV, the helper virus is simian sarcoma-associated virus (SSAV) that has an intact *env* gene [89,90,91,92]. Several other viral species also require helper virus complementation to complete their infection cycle [93,94,95]. The need for a helper virus implies that SSV is an evolutionary dead end unless it can regain a functional *env* gene by recombination. Sooner or later, SSAV will become so evolutionarily diverged from SSV that it can no longer serve as the helper virus for SSV. At that point, SSV will perish, and humans will lose an oncogenic agent unless SSV invades the germ cell line, which would ensure its existence as an endogenous retrovirus (i.e., a retrotransposon).

**Figure 5:**
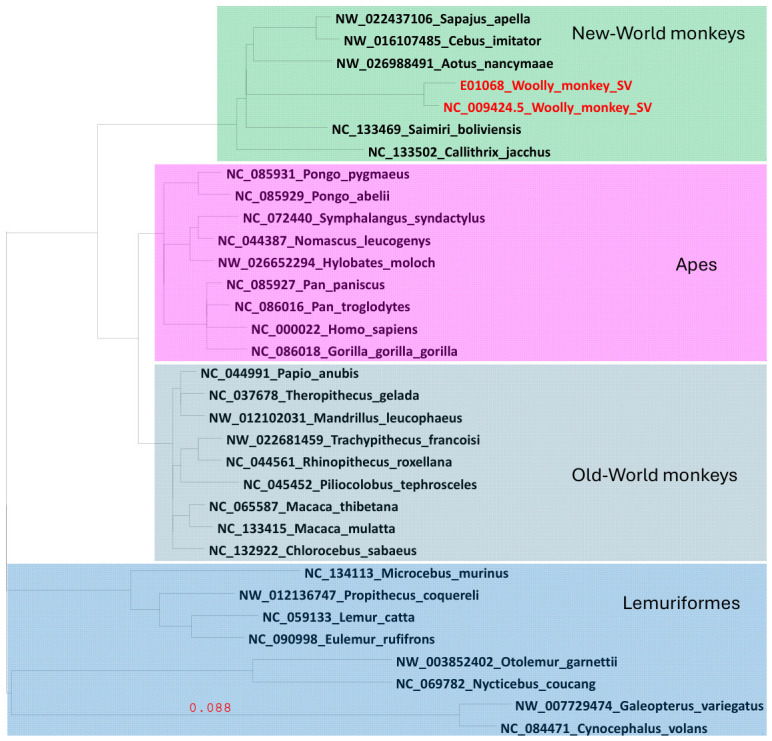
Phylogenetic tree of *PDGF-B* from major primate taxa and *v-sis* from woolly monkey sarcoma virus (colored in red). The phylogenetic reconstruction is based on aligned nucleotide sequences corresponding to sites 92–756 in Figure 4. Major taxonomic groups are shaded and labeled. Sequence names are in the form of “GenBank accession”_”species name”. One branch length (0.088 in red) was labeled to serve as a scale bar.

*PDGF-B* is present ubiquitously among primate lineages (Figure 5), as well as in other mammalian, avian, reptilian, and amphibian lineages [96]. The fact that PDGF and VEGF belong to the same gene family [97] suggests that PDGF could have originated early in vertebrate evolution. It is quite clear that *c-sis* (i.e., *PGDFB*) existed long before the origin of *v-sis* in SSV (Figure 5). The only two *v-sis* sequences available are closely related and form a monophyletic clade consistent with the interpretation of a recent origin (Figure 5). In short, the host-first hypothesis is strongly supported.

### 3.2. Retroviral Genes “Domesticated” by Host

Just like bacterial species that have coexisted with bacteriophages from time immemorial, eukaryotes have coexisted with retroviruses for perhaps billions of years. A retrovirus integrated in the host genome may get knocked out by mutations, lose its function to make infectious virions, and become an endogenous retrovirus, such as a retrotransposon or just a DNA “fossil” in the host genome [98]. Such inactivated or partially inactivated retroviruses on the host DNA that are no longer infectious are generally known as endogenous retroviruses (ERVs, being HERVs for human ERVs). The best studied HERVs are HERV-W [26,99], HERV-K [8,100,101,102,103], and HERV-FRD [104], each consisting of a group of retroviruses “fossilized” in human DNA through independent infection and decaying [8,105]. They are the decayed product of retroviral infection in the germline cells and are transmitted from parents to offspring like Mendelian genes. HERVs account for about 8% of the human genome [105] and could serve as the source of new genes and new traits for human or other retroviral hosts.

While retroviruses have borrowed many host genes [106], nature could also take the opposite direction by allowing the host to “domesticate” retroviral genes. One may argue that such domestication is unlikely because retroviruses almost always feature a set of genes with fixed functions, such as *gag*, *gag-pol*, *env*, etc., that would not be of much use for host cells. In contrast, vertebrate genomes feature a huge gene repertoire to serve as the source gene pool for retroviruses. Thus, one should expect to see more host genes used by retroviruses through transduction events than the other way round. However, there are several examples of the host “domesticating” retroviral genes, which have changed the early perception of ERVs as junk DNA [107,108].

#### 3.2.1. Mammalian Syncytin-1 and Its Origin

Human syncytin-1 is a protein encoded by a gene of retroviral origin (*HERV-W*) that has been co-opted by some mammalian clades to mediate cell–cell fusion during placental development, specifically contributing to the formation of the syncytiotrophoblast layer that regulates nutrient and gas exchange between mother and fetus [26,109,110,111,112]. The protein acts as a fusogenic envelope (env) glycoprotein, originally derived from an endogenous retrovirus (HERV-W). Most retroviruses use tRNA or tRNA-like sequences as primers for reverse transcription [113,114]. Because HERV-W most likely uses tRNA^Trp^ as its primer for reverse transcription, the letter W (one-letter code for Tryptophan) was appended to HERV in its classification [99,115], although the tRNA actually used could be tRNA^Arg^ [116].

The localized expression of Syncytin-1 was established through in situ hybridization and immunohistochemical assays, demonstrating that Syncytin-1 is specific to trophoblast cells, particularly in the multinuclear syncytiotrophoblast layer [26,109,110,111,112]. Its fusogenic function was confirmed using cell fusion assays, in which the gene, when expressed in cultured cells, induced membrane fusion characteristic of trophoblast syncytialization [117]. This function in normal cells is achieved by syncytin-1 binding to its ASCT2 receptor [118]. Thus, the sequence and functional similarities between mammalian Syncytin-1 and retroviral env proteins have been established beyond doubt. However, these similarities do not contribute to the discrimination between the host-first and retrovirus-first hypotheses on the origin of Syncytin-1.

The retrovirus-first hypothesis is assumed for the origin of syncytin-1, i.e., the gene originated first in retroviruses and was “domesticated” by the host. Two lines of evidence were presented in support of the retrovirus-first hypothesis. First, the *ERVW-1* gene (encoding syncytin-1) was identified when the retroviral *env* gene sequence of an endogenous retrovirus HERV-W was used to scan the human genome for matches [26]. Second, the *ERVW-1* gene appears to be located at the position of the retroviral *env* gene within a remnant of an HERV. The first line of evidence of sequence similarity between mammalian *ERVW-1* and retroviral *env* does not actually contribute to the discrimination between the two hypotheses. The second line of evidence would lend direct support for the retrovirus-first hypothesis, but the evidence cannot be clear-cut because of mutations obliterating the identity of flanking sequences. In fact, the original paper assuming the retrovirus-first hypothesis [26] did not present this second line of evidence.

An alternative approach to support the retrovirus-first hypothesis for the origin of syncytin-1 is to see if the genomic region flanking the *ERVW-1* gene (encoding syncytin-1) is a retrovirus integration hotspot [109]. The human *ERVW-1* gene is in the following configuration in the genome: *PEX1*…*ERVW-1*…*ODAG*. Bonnaud et al. [109] amplified the sequences between *PEX1* and *ODAG* in 14 mammalian and two avian species. This intervening sequence varies much in length, which is mainly due to the insertion and deletion of transposable elements [109]. This finding increases the plausibility that a retrovirus colonized the region and provided the original *env* gene for domestication.

**Figure 6:**
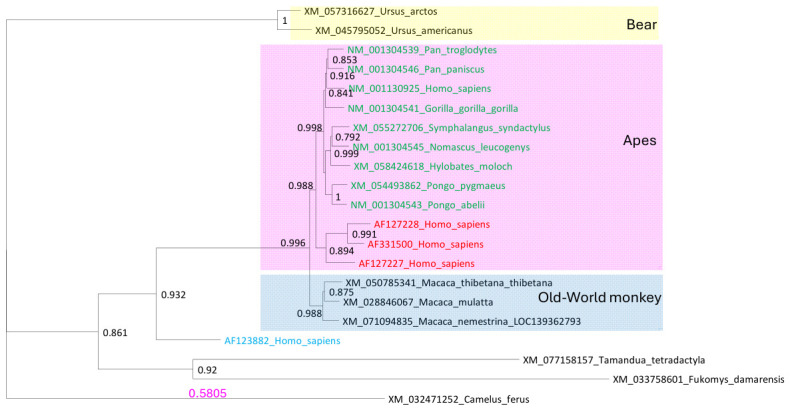
Phylogenetic tree of all orthologous *ERVW-1* encoding syncytin-1 from the NCBI Orthologs database, plus four sequences from human MSRV (multiple sclerosis-associated retrovirus, with three colored red and one colored blue), all being derived from the *env* gene of ERV-W lineages. The phylogenetic reconstruction is based on aligned coding sequences. Major taxonomic groups are shaded and labeled. Sequence names are in the form of “GenBank accession”_”species name”. Bootstrap support values greater than 0.5 are labeled next to the node. One branch length (0.5805 in pink) was labeled to serve as a scale bar.

The phylogenetic tree (Figure 6) of aligned sequences of syncytin-1 plus four sequences from multiple sclerosis-associated retroviruses (MSRV) shows sequence divergence patterns typical of horizontal gene transfer. First, the tree does not reflect genealogical relationships among the mammalian species. For example, five human homologues (four colored red and one lustered with chimpanzee and gorilla) are highly diverged from each other instead of all being clustered with chimpanzees as sister lineages. The simplest explanation of this pattern requires the occurrence of three independent transduction events involving three different viral lineages: (1) occurring in the common ancestor of the apes (colored green in Figure 6) and having become the functionally important syncystin-1 gene, (2) occurring in the common ancestor of the three MSRV lineages (colored red in Figure 6), of which one is a recombinant [119], and (3) occurring in a human lineage colored blue (Figure 6) and evolving into an extra MSRC lineage. Second, the sequence divergence in the tree of syncytin-1 and its homologues in Figure 6 is greater than one would expect from an average mammalian gene. For example, the human MSRV lineages colored red in Figure 6 have an evolutionary distance of about 0.35 from the human MSRV lineage colored blue in Figure 6. This could have two explanations. The first is the independent occurrence of transduction events involving two highly diverged viral lineages. The alternative explanation invokes an ancient gene duplication event followed by the loss of one paralogue in all primate species, except for humans, in Figure 6. This explanation would require many independent losses of the paralogs, which should be highly improbable. Thus, the sequence divergence between the red and the blue MSRV lineages in Figure 6 reflects the sequence divergence of two viral lineages before their independent transduction events. That is, the sequence divergence does not represent divergence within humans after a single transduction event. The evolutionary rate is about 2.2 × 10^−9^ substitutions/site/year for mammalian nuclear genomes [120], but can reach 0.003 or even greater for SARS-CoV-2 [121]. Retroviruses typically do not have a proof-reading enzyme, so their evolutionary rate could be even higher.

Previous studies have shown that different syncytin genes arose from independent “domestication” events. Figure 6 shows that even the same syncyctin-1 in different mammalian lineages arose from multiple independent “domestication” events. Leaving out the four sequences from MSRV, one would need five such independent “domestication” events to explain the phylogenetic pattern, i.e., one for the bear species, one for the (Ape + Old-World monkey) clade, and one for each of the three species at the bottom of the phylogeny in Figure 6. This is consistent with the previous findings that these *ERVW-1* genes are located in the *env* region of the endogenous retroviruses at different decaying stages in mammalian genomes [26,99,109,110,111,112,115].

The four sequences from MSRV were from multiple sclerosis patients [103,119]. They apparently are from two independent endogenization events, but neither leads to syncytin-1. Thus, the *env* gene of an ERV could result in a useful protein such as syncytin-1, but could also become something potentially contributing to multiple sclerosis, although the link between ERV and multiple sclerosis has not been fully established [122]. Multiple sclerosis is an autoimmune disease that occurs when our immune system has difficulty distinguishing between self and non-self. ERVs are the key culprit confusing the immune system. In terms of genome residency, ERVs are clearly “self” because they are a permanent fixture of our DNA. In terms of behavior, ERVs are “non-self” because they are descended from retroviruses.

Syncytin-1 is present in apes, Old-World monkeys, and some rodent lineages, but it is absent in several other mammalian clades, including carnivores (e.g., dogs and cats), ruminants (e.g., cows and horses), and marsupials [123]. These taxa instead possess entirely different syncytin-like genes (e.g., syncytin-A and syncytin-B in mice) [124], demonstrating that the capture of a fusogenic retroviral env gene has occurred multiple times independently during mammalian evolution.

The sequences in Figure 6 do not match any current exogenous retroviruses. The progenitor of the *env*-derived sequences in Figure 6 must have either perished or evolved beyond recognition. In short, the evidence is in favor of the retrovirus-first hypothesis.

#### 3.2.2. Mammalian Suppressyn and Its Origin

Like syncytin-1, suppressyn, encoded by the *ERVH48-1* gene, was co-opted from the *env* gene of ERV-F [118], where the letter F stands for the priming tRNA^Phe^. Suppressyn is a protein that serves two functions. The first function is related to syncytin-1 in the previous section that maintains the optimal oxygen and nutrient supply to the fetus through its fusogenic function, achieved by the ASCT2/SLC1A5-mediated cell fusion. Suppressyn competes for the same receptor to suppress cell fusion [118,125]. The expression of suppressyn depends on oxygen and Fe^2+^ concentration, mediated by HIF1α. At normoxia and a normal Fe^2+^ level, HIF1α is degraded through the ubiquitination pathway and consequently has no effect on suppressyn expression. At hypoxia and a low Fe^2+^ level, HIF1α is stabilized and enters the nucleus to form a heterodimer with HIF1β. The heterodimer binds to the HRE (hypoxia response element) of the *ERVH48-1* gene encoding suppressyn and upregulates the expression of suppressyn [125]. This might appear counterintuitive. In hypoxia, there should be more syncytialization to facilitate oxygen supply, so there should be less suppressyn expression. The possible explanation is that suppressyn is needed to prevent over-syncytialization at the wrong places. Thus, although both syncytin-1 and suppressyn are expressed in the mammalian placenta, there should be fine-scale spatial differences in their expression.

The second function of suppressyn is to defend host cells against type D retroviruses [126]. These retroviruses bind to the ASCT2/SLC1A5 receptor to initiate cell fusion and viral entry. By binding to the same ASCT2/SLC1A5 receptor, the viral entry through cell fusion is prevented [118,125]. Different RNA viruses use different mechanisms to promote cell–cell fusion. For example, SARS-CoV-2 uses ACE2, furin, and TMPRSS2 to promote cell–cell fusion [127].

**Figure 7:**
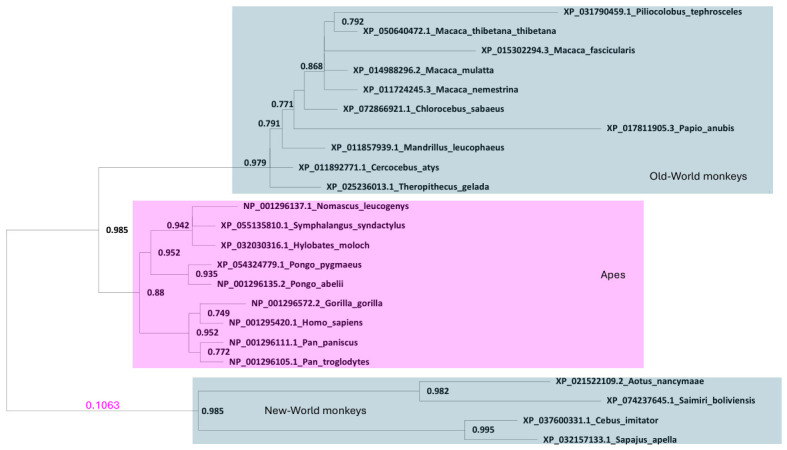
Phylogenetic tree of all 23 mammalian orthologous *ERVH48-1* encoding suppressyn from the NCBI Orthologs database. The phylogenetic reconstruction is based on aligned amino acid sequences. Major taxonomic groups are shaded and labeled. Sequence names are in the form of “GenBank accession”_”species name”. Bootstrap support values greater than 0.5 are labeled next to the node. One branch length (0.1063 in pink) was labeled to serve as a scale bar. The tree was mid-point-rooted to facilitate visualization.

Two lines of evidence are relevant to support the retrovirus-first hypothesis for the origin of suppressyn, i.e., the *ERVH48-1* gene (encoding syppressyn) was derived from the retroviral *env* gene. Two lines of evidence would substantiate this hypothesis. The first is to check if the *ERVH48-1* gene is located within the remnant of a retrovirus, at the location where the env gene should be. The second is to perform a phylogenetic analysis to see if the phylogenetic distribution of *ERVH48-1* is consistent with horizontal gene transfer. The first line of evidence was partially provided by the observation that the *ERVH48-1* gene has a promoter within a sequence homologous to a retroviral 5′ LTR sequence [118,125]. The second line of evidence appears to contain an error. It was claimed that the *ERVH48-1* gene is present only in apes and Old-World monkeys but not in other primate lineages, in particular, not present in New-World monkeys. As the common ancestor of Simiiformes (apes + Old-World monkeys) originated ~27–38 million years ago, it was inferred that the primate *ERVH48-1* gene was domesticated from an ancient retroviral *env* gene around that time. As one can see from Figure 7, the *ERVH48-1* gene is, in fact, present in New-World monkeys. However, this does not mean that a single gene-domesticating event occurred at the origin time of the common ancestor of the Old-World monkeys and the New-World monkeys, which is about 40.0–44.2 million years (median time) [128]. Instead, the tree in Figure 7, as explained below, suggests multiple independent gene-domesticating events in various primate lineages.

There are currently 23 mammalian suppressyn orthologs in the NCBI Orthologs database. The phylogenetic tree (Figure 7) again shows a strong signature of horizontal gene transfer. For example, *Cebus imitator, Sapajus apella*, and *Saimiri boliviensis* are more closely related to each other, all belonging to the family Cebidae. However, the tree in Figure 7 clustered *Saimiri boliviensis* with *Aotus nancymaae* (belonging to a different family, Aotidae). This is most likely caused by nearly identical retroviruses infecting both *S. boliviensis* and *A. nancymaae* and becoming domesticated independently. Thus, their phylogenetic affinity reflects the retroviral genetic affinity instead of the nuclear sequence divergence since speciation. The same explanation can be applied to the clustering of the two *Pongo* species with gibbons instead of with other great apes. Similarly, among the Old-World monkeys, the genera *Papio, Macaca*, and *Mandrillus* are in the Papionini tribe, but *Chlorocebus* belongs to a different tribe (Cercopithecini). However, the *ERVH48-1* tree (Figure 7) mixed all of them together. The very long branch lengths leading to *Piliocolobus tephrosceles* and *Papio anubis* suggest independent horizontal transfer events, i.e., two divergence retroviruses independently endogenized in these two primate species. All these can be simply explained by horizontal gene transfer, but are difficult to explain with vertical gene transfer. In short, the phylogeny supports the retrovirus-first hypothesis for the origin of suppressyn.

## 4. Discussion

Nature has been a creative tinkerer. Retroviruses can mine a host gene to increase their fitness, and the host of the retroviruses can co-opt a retroviral gene to increase its fitness. When a retroviral genome resides on the host genome after reverse transcription and integration, its only way of increasing its fitness is to encourage the host cell to replicate fast so that the viral genome can be replicated as a hitchhiker. Both *v-Src* and *v-sis* were mined from the host genome by the retroviruses to increase host cell replication. From a viral perspective, an ideal host cell is one that can rapidly replicate out of control. From a host perspective, such out-of-control replication would lead to cancer. The battle between the host and the retroviruses has left behind many retroviral “corpses”, i.e., ERVs, in the host genome. Are these endogenous retroviruses true war victims or dormant tumorigenic agents that could be revised [8]?

The transduction of a host gene by a retrovirus represents a major mutation event on the viral genome. Many different host genes could be incorporated into the retroviral genome [22]. Most of these incorporation events are expected to be deleterious to the retrovirus when the incorporated host gene sequence carries no benefit but a high cost of disrupting an essential retroviral gene. Nature generally does not leave such unfortunate viruses for us to observe because they are immediately purged from the retroviral population, although defective viruses are occasionally observed before their disappearance [94].

Occasionally, the incorporation of a host gene sequence into a retroviral genome carries both a benefit and a cost, e.g., the beneficial *v-sis* from the host coupled with the cost of a disrupted *env* gene and replication-incompetence in SSV. The benefit of such a mutation leading to a replication-incompetent SSV is obvious when it arises from a population of replication-competent SSAV with an intact *env* gene [89,90,91]. This rare SSV gains the benefit of increased replication rate through the function of *v-sis* but does not suffer from the cost of replication-incompetence because of the abundance of SSAV. However, when SSV increases in frequency at the cost of SSAV, the cost of replication-incompetence will manifest, so the relative abundance of SSV and SSAV will reach a balance through this frequency-dependent selection. Even among RSV, there are replication-competent and replication-incompetent strains. The Prague-C strain of RSV has its *v-Src* inserted in a region without essential retroviral genes, so it is replication competent. However, other RSV strains, such as RSV-29 [88] and two clonal variants of the Bryan strain of RSV, RSV(-)3, and RSV(-)16 [95] have gained the *v-Src* gene at the cost of losing a functional *env* gene and become replication-incompetent. Such evolutionary events provide excellent opportunities to study frequency-dependent selection.

One may imagine that a population of replication-incompetent retroviruses (e.g., SSV) may be so successful in infecting host cells and producing tumors that they reduce the replication-competent retroviruses (e.g., SSAV) to such a low level that the latter drift to extinction. When this happens, then the replication-incompetent retroviruses, after consuming all env proteins left over by the replication-competent ones, would go extinct as well, leading to an interruption and termination of cancer development. This could explain cases of spontaneous remission of cancer observed frequently by medical professionals [129,130,131].

Just like humans may harvest organs from corpses, eukaryotic hosts frequently harvest genes from ERVs. Introducing a stranger into one’s family is likely deleterious in most cases, even if the stranger might be willing to do some house chores. Syncytin-1 and suppressyn represent examples of successful domestication. However, even such successful cases are not without risk [112]. For example, abnormal cell fusion induced by syncytin-1, especially cell fusion between cancer cells and other cell types [132,133], could contribute to tumor heterogeneity, metastasis, and drug resistance. Interaction between syncytin-1 and other cancer-related genes or signal pathways, such as Wnt/β-catenin signaling, could also contribute to cancer development [134]. The same risk also exists for suppressyn [112].

What is the selective advantage associated with the genetic exchange between retroviruses and their hosts? For retroviruses that gained v-Src and v-sis, the selective advantage is obvious. That is, the two proteins stimulate cell proliferation of the host and consequently increase the fitness of the provirus that carry these genes. The selective advantage of syncytin-1 and suppressyn is not as direct. The “domestication” of syncytin-1 and suppressyn would have been much more significant if the domestication events occurred during the evolutionary transition from non-placental to placental mammals, i.e., they drove the evolutionary transition. Unfortunately, the origin of syncytin-1 and suppressyn did not occur that early (otherwise the placental mammals would tend to share these two genes and have a syncitin-1/suppressyn tree concordant with the species tree). As is, the two genes only function to refine the placental system, not to create the system. This refinement is the first proposed selective advantage, i.e., there is no other gene pair that maintains the homeostasis in syncytialization. The second proposed selective advantage is to enhance the host defense against other retroviruses, especially Type D retroviruses, which has been substantiated experimentally [118,125,126]. However, we do not know if the ancient mammals were exposed to Type D retroviruses or if Type D retroviruses significantly reduce the fitness of mammals.

## 5. Conclusions

Retroviruses, after integrating their genomes into those of host cells, replicate passively through host cell division. During this “hitchhiking” phase, their evolutionary success depends largely on promoting rapid and unregulated proliferation of the infected host cell. A virus that can “borrow” a host gene to promote host cell growth and replication is therefore evolutionarily beneficial to the virus. Both the *v-Src* gene of Avian sarcoma virus and the *v-sis* gene of Simian sarcoma virus were originally captured from host genomes. Their cellular counterparts, c-Src (a non-receptor tyrosine kinase) and c-sis (platelet-derived growth factor), normally regulate cell growth and signaling, but their viral derivatives drive uncontrolled cellular proliferation that can ultimately lead to cancer. The host, however, can also “borrow” viral genes to enhance the host’s fitness. Notable examples include syncytins, whose fusogenic activity is essential for trophoblast fusion and syncytium formation during placental development, and suppressyn, which both regulates syncytialization and contributes to resistance against retroviral infection. The observed similarity in sequence and in function typically leads to two hypotheses: (1) the retrovirus-first hypothesis, i.e., the gene originated in retroviruses but was domesticated by the host, and (2) the host-first hypothesis, i.e., the gene originated in the host and was co-opted by retroviruses. Molecular phylogenetics has become an indispensable tool for reconstructing the evolutionary history of these genetic exchanges between retroviruses and their hosts and discriminating between the two alternative hypotheses.

## Data Availability

The sequence files needed to replicate the results are in the zipped file SequenceFiles.zip.
